# Seascape Genetics and the Spatial Ecology of Juvenile Green Turtles

**DOI:** 10.3390/genes11030278

**Published:** 2020-03-05

**Authors:** Michael P. Jensen, Mayeul Dalleau, Philippe Gaspar, Maxime Lalire, Claire Jean, Stéphane Ciccione, Jeanne A. Mortimer, Mireille Quillard, Coralie Taquet, Andrew Wamukota, Géraud Leroux, Jérôme Bourjea

**Affiliations:** 1 Section of Biology and Environmental Science, Department of Chemistry and Bioscience, Aalborg University, Fredrik Bajers Vej 7H, 9220 Aalborg East, Denmark; michael@mpj.eu; 2 Centre d’Etude et de Découverte des Tortues Marines (CEDTM), 6 Chemin Dubuisson, Appt. 5, 97436 Saint-Leu, La Réunion, France; mayeuldalleau@cedtm-asso.org; 3 Mercator Ocean International, Parc Technologique du Canal, 31520 Ramonville Saint-Agne, France; pgaspar@mercator-ocean.fr; 4 Collecte Localisation Satellite, Sustainable Management of Marine Resources, Parc Technologique du Canal, 31520 Ramonville-Saint-Agne, France; mlalire@groupcls.com; 5 Kelonia, L’Observatoire des Tortues Marines, 46 Rue du Général de Gaulle, 97436 Saint Leu, La Réunion, France; claire.jean@museesreunion.re (C.J.); stephane.ciccione@museesreunion.re (S.C.); 6 Seychelles Islands Foundation (SIF), Mont Fleuri, Victoria, Mahé P.O. Box 853, Seychelles; jeanne.a.mortimer@gmail.com; 7 Department of Biology, University of Florida, Gainesville, FL 32611, USA; 8 Conseil Départemental de Mayotte/DEDDE/SE 8 Boulevard Halidi Selemani BP101, 97600 Mamoudzou, France; mireille.quillard@cg976.fr; 9 Collège de Hao, HAO 98767, Tuamotu Archipelago, French Polynesia; coralie.taquet@gmail.com; 10 Department of Environmental Sciences, Pwani University, P.O. Box 195, Kilifi 8010, Kenya; awamukota@gmail.com; 11 Muséum D’histoire Naturelle de Genève, Route de Malagnou 1, 1208 Genève, Switzerland; geraud.leroux@freesurf.ch; 12 MARBEC, Univ Montpellier, CNRS, Ifremer, IRD, Avenue Jean Monnet, 34200 Sète, France; 13 IFREMER Institut Français pour l’Exploitation de la Mer, Avenue Jean Monnet, 34200 Sète, France

**Keywords:** *Chelonia mydas*, green turtle, juvenile, mtDNA, drifting simulation, connectivity, mixed stock analysis, Southwest Indian Ocean

## Abstract

Understanding how ocean currents impact the distribution and connectivity of marine species, provides vital information for the effective conservation management of migratory marine animals. Here, we used a combination of molecular genetics and ocean drift simulations to investigate the spatial ecology of juvenile green turtle (*Chelonia mydas*) developmental habitats, and assess the role of ocean currents in driving the dispersal of green turtle hatchlings. We analyzed mitochondrial (mt)DNA sequenced from 358 juvenile green turtles, and from eight developmental areas located throughout the Southwest Indian Ocean (SWIO). A mixed stock analysis (MSA) was applied to estimate the level of connectivity between developmental sites and published genetic data from 38 known genetic stocks. The MSA showed that the juvenile turtles at all sites originated almost exclusively from the three known SWIO stocks, with a clear shift in stock contributions between sites in the South and Central Areas. The results from the genetic analysis could largely be explained by regional current patterns, as shown by the results of passive numerical drift simulations linking breeding sites to developmental areas utilized by juvenile green turtles. Integrating genetic and oceanographic data helps researchers to better understand how marine species interact with ocean currents at different stages of their lifecycle, and provides the scientific basis for effective conservation management.

## 1. Introduction

For many marine species, population connectivity is determined largely through the transportation of eggs, larvae and juveniles between different habitats by ocean currents [1,2], which also carry nutrients and contaminants, while influencing physical parameters such as temperature and salinity, all of which shape marine ecosystems. Technological advances in tracking, remote sensing and ocean modeling have expanded our understanding of the biophysics of marine dispersal, and its importance to population dynamics [3,4,5]. Recent work has focused upon understanding how ocean currents impact the distribution and connectivity of marine species, such as coral [6] and reef fish [7,8], but also larger marine migratory species, including sharks [9], whales [10] and turtles [11]. Understanding how marine species interact with ocean currents at different stages of their lifecycle provides the scientific basis for effective conservation management. 

After emerging from their nests onto tropical and subtropical beaches around the world, green turtle hatchlings (*Chelonia mydas*) enter the sea and are dispersed by ocean currents during the first several years of their lives, while drifting in pelagic habitats. The young turtles mature and grow in size, and upon reaching a curved carapace length (CCL) of about 35 cm, they settle into neritic feeding areas [12,13]. They typically show strong fidelity to a feeding area before reaching adulthood, or may move through a succession of developmental foraging sites [12]. Upon reaching adulthood, female green turtles usually demonstrate strong philopatry, as they migrate back to their natal region to breed. Adult foraging aggregations often comprise turtles from multiple genetic stocks [14,15], with some turtles migrating to local rookeries less than 100 km away, and others traveling several thousand kilometers to more remote breeding grounds [16,17]. The migratory pathways used by adult turtles are becoming well understood from satellite telemetry studies [18,19].

In contrast to the adult turtles, little is known about the spatial ecology of post-hatchling sea turtles, even if very recent advances in satellite tracking technology have allowed tracking post-hatchling turtles as small as 10 cm CCL [20]. These studies have shown that while post-hatchling turtles follow major ocean currents, they are able to actively swim away from unfavorable environments when they need to. While pelagic dispersal is known to be governed by ocean currents, the mechanisms causing immature turtles to settle into neritic foraging areas are less clear. The natal origins or stock of juvenile foraging aggregations, however, can be assessed through the use of genetic markers [15]. 

The maternally inherited mitochondrial (mt)DNA control region sequences have been used extensively in sea turtles [21], and elucidate the connectivity between nesting and foraging areas [22,23]. When the genetic stock structure of breeding populations are known (and sufficiently differentiated), they can be used as a reference to trace back the natal origin of turtles using a mixed stock analysis (MSA) [24,25]. This method can be applied to all age classes, and remains the only way to rapidly assess the connectivity between juvenile turtles and their natal origins. 

However, numerical simulations can provide a better understanding of the dispersal of juvenile sea turtles and the connectivity between nesting and foraging habitats. Dispersal patterns can be inferred by analyzing large numbers of simulated trajectories with computed trajectories, based on the assumption that hatchlings (and then juveniles) released from natal beaches drift passively with ocean currents. This simple approach has been, and is still widely used, to obtain a first-order estimate of the pelagic juveniles’ spatial and temporal distributions (e.g., [26,27,28,29]). More elaborate models have recently been developed [30,31] that simulate dispersal under the combined effect of ocean currents and active swimming movements motivated by the need to find suitable habitats (i.e., adequate water temperatures and food). Results of these models show that, while ocean currents broadly shape dispersal pathways, habitat-driven movements strongly modulate the spatial and temporal distribution of juveniles along these pathways. 

To date, these new active dispersal models have improved our understanding of the pelagic distribution of a few juvenile leatherback [5,31] and loggerhead [30] populations. Unfortunately, because such models have not yet been adapted for green turtles, a more usual passive drift model will be used here. Given that broad-scale dispersal is governed primarily by ocean currents, this simple approach is likely sufficient, in combination with genetic results, to unravel the oceanic connectivity of the early life stages of the green turtle. Previous studies combining genetic analyses and drift simulations have already shown the complex impacts that ocean currents can have on genetic connectivity [32,33,34]. Understanding this connectivity between distant populations, and how they mix at foraging sites, is key to effective conservation and management. 

The Southwest Indian Ocean (SWIO) is home to some of the world’s most unique terrestrial and marine biodiversity and ecosystems. It is considered a biodiversity hotspot [35], and hosts five species of sea turtles, which are found throughout the region [36]. Of these, the green turtle is the most abundant and widespread, and nests primarily on isolated islands, namely: the French Eparses Islands (Europa, Tromelin, Glorieuses, Juan de Nova), Mayotte, Comoros, Seychelles (granitics, Amirantes, southern islands, including Aldabra) and the Chagos archipelago [37,38]. Some smaller, nesting populations are also found along the African mainland (Kenya, Tanzania and Mozambique) and the coastline of Madagascar [36]. Previous work has characterized the genetic population structure of breeding populations throughout the SWIO region. Using mtDNA control region sequences from 15 rookeries, at least three separate genetic stocks (or management units) were identified spanning the entire SWIO [39]. These three genetic stocks each include several rookeries, and are situated in the South (Europa and Juan de Nova), Central (Tromelin, islands in the north Mozambique Channel, and rookeries along the coast of Kenya and Mozambique) and North (rookeries in the Seychelles granitics and Amirantes) of the SWIO. Satellite telemetry has shown that adult female turtles nesting on islands in the SWIO primarily utilize coastal foraging areas along the coasts of Madagascar and East Africa, often traveling thousands of kilometers between breeding and feeding sites [16,18]. Juvenile green turtles are also found at most adult breeding sites, and forage in large numbers in areas adjacent to the many islands and atolls in the region (e.g., [40,41,42]). Better understanding of the connectivity between juvenile developmental areas and regional breeding populations is critically needed for effective conservation management. Here, a combination of molecular genetics and particle simulations are used to elucidate linkages between juvenile turtles that utilize the vital developmental habitats and the nesting populations from which they originate. The specific aims of this study are to integrate the genetic results from MSA with simulations of ocean dispersal patterns to address the following questions within the SWIO region: (1) What are the levels of connectivity between nesting and developmental areas in the region? (2) Does the genetic structure of turtles at developmental sites vary with latitude?; and (3) How do patterns of connectivity patterns relate to ocean currents?

## 2. Material and Methods

### 2.1. Genetic Data Collection and Analysis

#### 2.1.1. Sample Collection 

**Figure 1:**
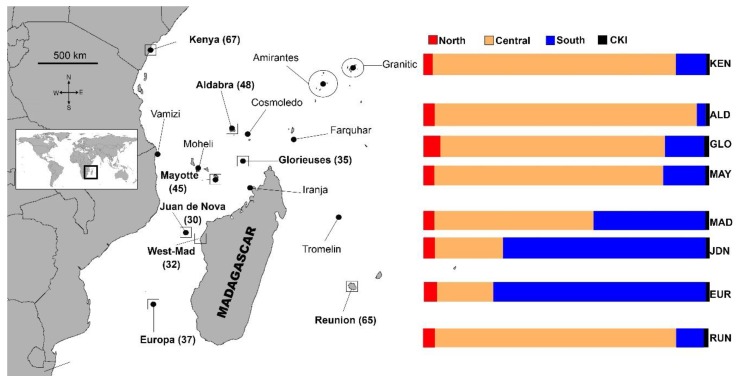
Location map. Locations of 15 green turtle (*Chelonia mydas*) nesting locations and eight foraging sites included in the mixed stock analysis (MSA). Black dots represent individual rookeries, and shaded squares define three genetically distinct groupings (stocks) used as our baseline. Foraging locations are represented by squares. The bar graph shows the mean relative contribution of **North**; *Granitics and Amirantes,* **Central***; Kenya, Aldabra, Cosmoledo, Vamizi, Mohéli, Mayotte, Glorieuses, Iranja and Tromelin;* **South***; Juan de Nova and Europa* and *CKI*, *Cocos (Keeling) Islands*, to each of the eight developmental sites (CKI not shown on the map). The 95% confidence interval (CI) can be found in Table 2. Estimates are based on the mixed stock analysis using the many-to-many package in “R”, and using the population size as weighted priors.

Tissue samples were collected from juvenile green turtles with curved carapace lengths < 80 cm (mean = 53.2 cm, standard deviation (SD) = 11, *n* = 359; see Table S2 for details), at eight developmental sites across the SWIO, between 1.5 and 40°S, and 39 to 56° E (Figure 1, Table 1 and Table 2). Six sites are isolated islands, including five in the French Overseas Territories: Europa (*n* = 38), Juan de Nova (*n* = 24), Glorieuses (*n* = 31), Mayotte (*n* = 53) and La Réunion (*n* = 65); and Aldabra Atoll Seychelles (*n* = 47). Two sites are located adjacent to larger land masses: Kenya (between Mida Creek and Lamu) (*n* = 67); and west Madagascar (Maintirano area) (*n* = 33). At the island sites, juvenile turtles were captured, either in shallow waters using the rodeo method of jumping onto them from a small boat [43], or in deeper waters by scuba divers. At Kenya and west Madagascar, samples were collected from both living and dead individuals taken as by-catch in the artisanal fishery. 

Skin samples were collected using either a sterilized scalpel or a 6 mm biopsy punch, stored in 20% dimethyl sulfoxide (DMSO) buffer saturated salt solution [44] or 70% EtOH, and frozen until DNA extraction. More details on sampling are available in Taquet [45]. 

All samples were collected under the ethical approval code DA 2014-03 and project authorization (09-14-05/SG/DRCTCV), provided by La Réunion Environment, Planning and Housing Direction (DEAL) from the Ecological and Solidarity Transition French Ministry (MTES). CITES import permit numbers by country are: Juan de Nova FR0597400138-I, Europa FR0597400137-I, Mohéli FR0597400133-I, Mayotte FR0597400132-I, west Madagascar FR0597400134-I and FR0597400135-I, Tromelin FR0597400140-I, Glorieuses FR0597400139-I, Seychelles FR1297400259-I and Kenya 2371-I-165067.

#### 2.1.2. DNA Extraction, Sequencing and Regional Data Compilation

Total genomic DNA was extracted from tissue samples using a salting-out method, and a 385 base pair portion of the mtDNA control region was amplified by polymerase chain reaction (PCR), using both the primers TCR-5 (5-TGTACATTACTTATTTACCAC-3) and TRC-6 (5-GTACGTACAAGTAAAATACCGTATGCC-3). All protocols followed those described in Bourjea et al. [46]. Briefly, amplifications were performed in a total volume of 25 μL, containing 5–50 ng of whole DNA, 0.2 mM of each dNTP, 10 μM of each primer, 1 U of high-fidelity Advantage 2 polymerase mix (BD Biosciences, San Jose, CA, USA), and the corresponding reaction buffer (1x). Cycling parameters were 93 °C for 1 min, followed by 35 cycles at 93 °C for 40 s, 55 °C for 50 s and 72 °C for 40 s, and a final extension at 72 °C for 2 min. PCR products were sequenced on a NGS GS-6FLX sequencer (Roche Diagnostics, Meylan, France). All sequences cleaning, alignment and analyses were done using Geneious 8.1 [47]. 

#### 2.1.3. Data Analysis

Haplotype nomenclature was standardized according to Southwest Fisheries Science Center (http://swfsc.noaa.gov/prd-turtles.aspx) for the 386 bp fragment, with Pacific and Indian Ocean haplotypes being assigned a CmP prefix [48], while haplotypes belonging to the Atlantic clades had been assigned the CM-A prefix. The genetic diversity of turtles at each developmental site was assessed by calculating the haplotype (Hd) and nucleotide diversity (π) [49] using DnaSP v6 [50]. A parsimony network of haplotype relationships was constructed using the software TCS 1.13 [51,52]. 

To determine the stock proportions of the turtles sampled at each developmental area, we applied a Bayesian mixed stock analysis (MSA) using two different approaches. The first involved the software BAYES [24] (extensively used in sea turtle studies); and the second used the “many-to-many” mixstock package in R [25]. Both approaches estimated the proportional contribution of each genetically distinct rookery region (stock) to each developmental site. However, the “many-to-many” approach differs from BAYES, in that it incorporates all stocks and all developmental sites into one analysis. Therefore, combining all stocks allows for both a foraging site-centric estimate (i.e., What is the proportion of stocks at each foraging site?) and a rookery-centric approach (i.e., What proportion of turtles from each stock goes to each foraging site?). 

For the stock baseline, we initially compared haplotype frequencies from published mtDNA frequencies globally [48]. A total of 38 genetic stocks from the Indian, West Pacific and South Atlantic shared at least one haplotype with the juvenile samples used in this study, including the three genetic stocks located within the SWIO [39]. The initial analysis was conducted using BAYES only (flat priors), and showed that distant stocks in Southeast Asia and the western Pacific were unlikely contributors (<3% contribution) apart from East Taiwan. However, East Taiwan is a small population located approximately 10,000 km away, and is fixed for haplotype CmP49, which is the most common haplotype in the SWIO. Therefore, any contribution from East Taiwan was considered highly unlikely, and not included in the final analysis (Figure S2). Also, the global MSA indicated significant contributions from the Cocos (Keeling) Islands stock, which is located centrally in the Indian Ocean. Therefore, possible contributions from the Cocos (Keeling) Islands were considered likely, and this stock was included in the final MSA. 

Finally, the global MSA also resulted in several stocks in the South Atlantic Ocean at proportions that were considered unlikely, and thus suspected of being a statistical artifact caused by widespread haplotypes (Figure S2). To assess the accuracy of the MSA in estimating contributions from the south Atlantic, we performed two simple simulation experiments. The first included Atlantic rookeries, as well as the three SWIO stocks and Cocos (Keeling) Islands. A simulated foraging sample of (*n* = 50) was made up of 75% South Area, 20% Central Area and 5% North Area, with 0% contribution from the Atlantic and Cocos (Keeling) Islands. The results from the MSA of the simulated dataset were then compared to the true (simulated) proportions. The purpose of this exercise was to see if the MSA would overestimate the contributions from the Atlantic. As expected, several Atlantic rookeries in this scenario were significantly overestimated. To make sure that these confounding issues were not affecting the MSA results within the SWIO, we performed a second simulation restricted to the three SWIO stocks and Cocos (Keeling) Islands. 

This simulation clearly showed that without the confounding factors of Atlantic rookeries, the MSA performs well in estimating the true (simulated) proportions (see Appendix C for more details). In addition, the Cape of Good Hope is a major biogeographical barrier, and ongoing green turtle connectivity between the Atlantic and Indian Ocean is probably rare (see our Discussion). For these reasons, the Atlantic stocks were omitted from the final MSA.

Based on the initial analysis, a reduced baseline was chosen that included only the four most likely sources: the South, Central and North SWIO stocks plus the stock from Cocos (Keeling) Islands. The final MSA was run using both BAYES and the “many-to-many”, using weighted priors. The priors were weighted relative to the size of the stock (mean annual number of females), under the assumption that larger stocks are more likely to contribute to a developmental site than smaller ones. Each MSA was run using four independent chains, with different initial distributions set at 0.925 for one stock, and 0.025 for each of the remaining stocks. Each chain was run with a burn-in of 25,000 steps, followed by 25,000 sampling steps. We used the Gelman and Rubin shrink factor diagnostic to assure that all chains had converged (shrink factor <1.2) [24].

### 2.2. Passive Drift Simulations

The passive dispersal of hatchlings and then juveniles was simulated for seven nesting beaches: two in the North Area (Seychelles granitic and Amirantes groups), four in the Central Area (Glorieuses, Mayotte, Mohéli and Tromelin) and one in the South Area (Europa). The technical setup of the simulations is identical to that of Gaspar et al. [27]. Individual trajectories are computed using the ARIANE Lagrangian trajectory simulation software (www.univ-brest.fr/lpo/ariane), fed with surface currents taken from the daily outputs of the GLORYS-1 (G1) reanalysis of the World Ocean circulation [42]. This reanalysis, performed with the NEMO numerical ocean model (www.nemo-ocean.eu), is provided by Mercator-Ocean (www.mercator-ocean.fr). The G1 model has an eddy-permitting horizontal resolution of 0.25° and 50 vertical layers. It covers the 7-year period going from 01/01/2002 to 31/12/2008. The G1 reanalysis assimilates satellite-derived sea level anomalies and sea surface temperature (SST) data, as well as in-situ temperature and salinity measurements. It proves to be especially well suited for simulating surface drifter trajectories [43].

To simulate the effect of the swimming frenzy in the first days, virtual hatchlings were released offshore of the nesting beaches. In the North Area, most of the islands of the Seychelles granitics and Amirantes groups host small nesting populations, so departures were simulated in two 0.25° × 0.25° areas central to each group. In the Central and South Areas, hatchlings were released in 0.25° × 0.25° areas located about 40 km off the main nesting beaches of Glorieuses, Mayotte, Mohéli and Europa islands. Release positions were uniformly distributed within the release area, and the numbers of releases per day follows a truncated normal distribution. Table 3 shows the dates of release in each area, which correspond to the peak hatching season at each nesting site [38] (i.e., peak nesting season plus the 2 months-long average incubation period). For each nesting site, the trajectories of 10,000 passive individuals were computed. For each individual released, daily positions were obtained over a one-year period. To account for the interannual variability of the oceanic circulation, such simulations were repeated for five consecutive years (2002 to 2006, inclusive). 

A total of 50,000 one-year-long trajectories of passively drifting individuals was produced for each nesting site. To ease the comparison of the modeled particles’ drifting patterns, we used a similarity index between trajectories from each pair of rookeries. For each rookery, we computed a density matrix as the number of particles recorded across the simulation in each cell of a 200 × 200 spatial grid between latitude 55° S and 30° N and longitude 15° W and 120° E. The number of daily positions (or “turtle days”) per grid cell was then used as a proxy for the turtle density in that grid cell. Therefore, we calculated the pairwise Euclidean distance (D) between the grids as follows, for the two density grids, G1 and G2, and each of the cells, g1 and g2:

(1) $$D\left(G_{1},G_{2}\right)=\sqrt{\underset{G_{1}}{\sum }\underset{G_{2}}{\sum }{\left(g_{2}-g_{1}\right)}^{2}}$$

Once we obtained each pairwise distance, we computed a heatmap summarizing the distance between each rookery, and clustered using an iterative clustering method, joining the two most similar clusters at each step, and continuing until there is just a single cluster (hclust function from R CRAN).

## 3. Results

### 3.1. Genetic Diversity

A total of 13 haplotypes were identified across all developmental areas (all Genebank ID are provided in Table S1). The most common haplotype was CmP49 (61%), followed by CM-A08 (18%) and CmP47 (11%), while the remaining ten haplotypes accounted for less than 10% of the total samples (0.3–3% each). The frequency of the most common haplotypes changed with latitude. Haplotype CM-A08 was the most common in the southernmost developmental sites, and accounted for approximately 58% and 50% of juvenile turtles at Europa and Juan de Nova, respectively, but decreased to account for only ~10% of haplotypes at more northern sites, and was absent from Aldabra (Table 1). The most common haplotype (CmP49), found at all developmental sites, increased in frequency towards the northern sites, and accounted for 81% of the haplotypes at Aldabra (Table 1). Three haplotypes were orphan, meaning that they have yet to be identified at a nesting rookery. Orphan haplotypes made up less than 3% of the total samples and 0–6% of samples at individual developmental sites (Table 2). Genetic diversity was similar for all developmental sites, with the lowest values found at Aldabra (Hd = 0.3333 and π = 0.0154) and the highest diversity found at Glorieuses (Hd = 0.6874 and π = 0.0328) (Table 1). 

The haplotype network (Figure S1) clearly showed that three highly divergent haplogroups are found among the genetic stocks in the region. Haplogroup 1 is mainly found at nesting sites in the South and Central regions, while haplogroups 2 and 3 are most frequent in the Central and North regions. It is also clear that orphan haplotypes found in this study are all closely related (1bp) to haplotypes found at rookeries within the region. 

### 3.2. Mixed Stock Analysis

The reduced baseline MSA showed that the two methods gave similar results, but with the “many-to-many” providing tighter confidence intervals (Table S3), hence only the results from the “many-to-many” MSA will be presented and discussed. Overall, the MSA suggested that most turtles from all eight feeding grounds originated from the three SWIO stocks (South, Central and North), and with the Cocos (Keeling) Islands (CKI) being likely, but at very low frequencies (Table 2). While the global MSA (flat prior) showed unreasonably high contributions from CKI, the reduced MSA using population size priors indicated smaller contributions in line with what we would expect from a distant and small (low 100 s females) nesting population [53]. 

**Figure 2:**
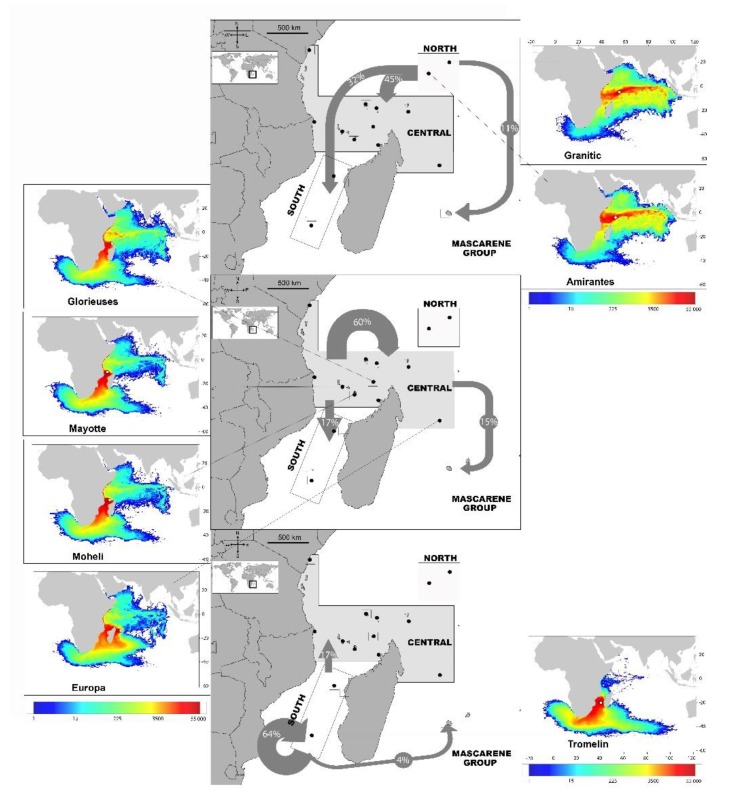
Dispersal from rookeries. Small maps show the density distributions of juveniles emerging from the seven simulated nesting areas (Colors indicate the number of particles). The three central maps show the proportional distribution of juvenile turtles from each Area (North, Central and South) based on Bayesian “rookery-centric” estimates using the many to many mixed stock analysis.

Rookeries belonging to the North stock contributed little to all feeding sites (mean 3–6%, Figure 1, Table 2). Overall, Central rookeries contributed the most, but there was a clear latitudinal gradient, with Central rookeries contributing the least at the southern developmental sites (Europa; mean = 20%, 95% CI = 1–42% and Juan de Nova; mean = 24%, 95% CI = 2–52%) and the most at the northern developmental sites (Kenya; mean = 85%; 95% CI = 70–95% and Aldabra; mean = 92%; 95% CI = 73–99%). The opposite pattern was seen for rookeries in the South which contributed the most to the southern sites (Europa; mean = 74%; 95% CI = 53–91% and Juan de Nova; mean = 71%, 95% CI = 43–94%)) and the least at the Kenya (mean = 11%; 95% CI = 2–22%) and Aldabra (mean = 3%; 95% CI = 0–11%) developmental sites. Being more geographically isolated than the other nesting and developmental sites included in the analysis, the developmental site at La Réunion mainly comprised turtles from the Central rookeries (mean = 85%, 95% CI = 63–96%) (Figure 1, Table 2). The “rookery-centric” MSA results show that the proportion of turtles from each rookery region go to each developmental site, as well as to “unknown” sites. The results estimated that most young turtles hatched in the South recruit into developmental areas in the South (64%), and less so to the Central (17%), La Réunion (4%) and unknown (15%) areas. Most juvenile turtles born in the Central Area also recruit to sites in the Central Area (60%), while the rest disperse to the South (17%), La Réunion (15%) and unknown (8%) areas. Finally, juvenile turtles from the North Area disperse to both the Central (45%) and South Areas (32%), as well as to La Réunion (11%) and unknown (12%) sites (Figure 2, Table S4). 

### 3.3. Passive Dispersal Simulations

Oceanic circulation in the Indian Ocean is complex and highly variable, especially in the northern part of the basin that is exposed to alternating monsoonal winds (See [54] for a full, detailed overview of ocean activity in the Indian Ocean and Appendix A for summary). 

Density distributions of juveniles emerging from the seven simulated nesting areas are shown in Figure 2 (and Appendix B). Dispersal patterns are fully consistent with the known circulation patterns in the west Indian basin. Each of the North, Central and South Areas appear to be associated with one specific dispersal pattern (Figure 2). In the North Area, simulated juveniles disperse essentially zonally in the equatorial band under the main influence of the South Equatorial Counter Current (SECC). 

The Somali current also disperses individuals along the African coast, both northward and southward, depending on the season, but in a rather limited latitude range, with dispersal into the Mozambique Channel particularly limited. Relatively high densities are only observed in the north-westernmost part of the Channel, west of the Comoros. 

In contrast, juveniles originating from the nesting beaches of the Central Area are mainly under the influence of the Northeast Madagascar Current (NEMC) and then the Somali Current (SC), with individuals dispersing northwards along the coast of Tanzania, Kenya and Somali. During the winter monsoon, part of these juveniles are entrained eastward by the SECC. Juveniles also disperse southwards, widely into the Mozambique Channel, where they are pushed by the Mozambique Current (MC). Finally, most juveniles originating from Europa (South Area) are carried southwards by the Mozambique and then the Agulhas Current. Southward mesoscale turbulences also induce limited northward dispersal, mostly into the central part of the Mozambique Channel. 

**Figure 3:**
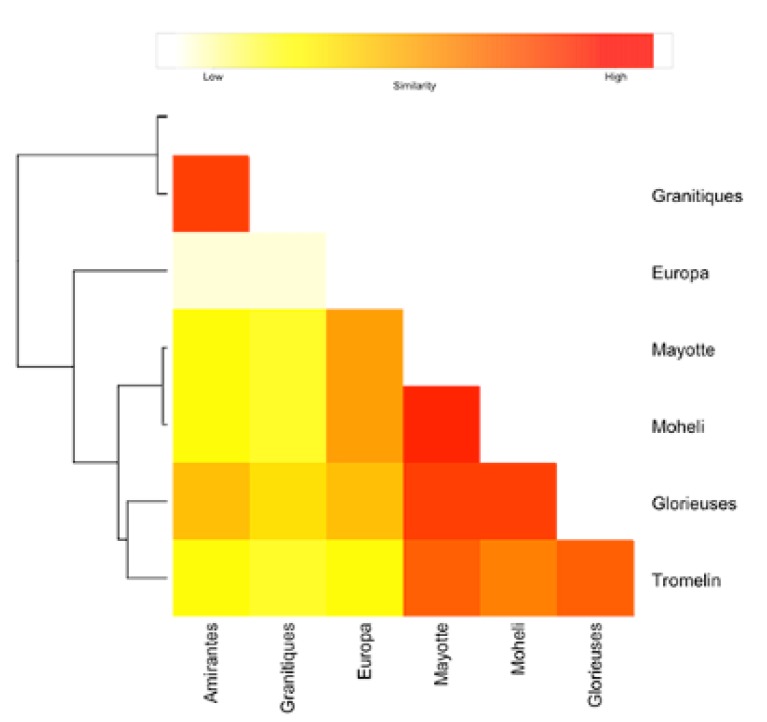
Schematic heatmap of pairwise similarity between drifting trajectories patterns of modeled particles from seven green turtle rookeries of the South West Indian Ocean. Light yellow/Intense red represents low/high similarity between drifting patterns. Drifting patterns similarities were computed as the opposite of the Euclidean distance between drifting density matrices.

More globally, the similarities, or differences, between the various juvenile density distributions and associated dispersal schemes, are shown in Figure 3, and confirm that:

1. The dispersal schemes (and corresponding density maps) associated with the nesting sites within the same Areas (North, Central and South) are similar.
2. The largest differences (smallest connectivity) are observed between the juvenile density maps of the South (Europa) and North (Seychelles granitics and Amirantes) Areas.
3. Nesting beaches of the Central Area display some connectivity with both the South and North Areas. As expected, Europa is more connected with Mayotte and Mohéli through the mesoscale eddy activity that mostly takes place on the western side of the Mozambique Channel. Connectivity with Glorieuses, lying further to the East, is more limited.

## 4. Discussion 

### 4.1. Source Rookeries Included in the Analysis 

In this study we used genetic analysis and hatchling dispersal simulations to investigate the spatial ecology of eight juvenile developmental areas, and to assess the role of ocean currents in driving the dispersal of green turtle hatchlings within the SWIO. While the initial MSA indicated a significant contribution from Atlantic rookeries, we excluded those rookeries from the Atlantic as potential sources, based upon two observations. Firstly, the contribution in the MSA was strictly driven by the haplotype CM-A08, which is shared between the Atlantic and the SWIO rookeries. No other Atlantic haplotypes were found, except CM-A46, which is extremely rare at Ascension Island, and only found in one individual at Europa. Secondly, previous oceanic current simulations suggests that frequent connectivity between the Atlantic and Indian Oceans is unlikely, given that all of the particles from the Atlantic that enter the Indian Ocean would drift eastward with the circumpolar current at latitude ~40° S in cold waters, and with a low probability of hatchling survival at those temperatures [55,56]. Nevertheless, the very small percentage of juvenile turtles coming from Atlantic nesting grounds that would approach the Cape of Good Hope are likely to be at least 4–5 years old [33], and therefore competent swimmers and more cold resistant, with a higher chance of survival than hatchlings. Thus, we cannot exclude that the combined actions of the circumpolar current and active movements of older individuals could allow the turtles to escape this cold current, and eventually reach the Mozambique Channel. Given the shared haplotypes between rookeries in these two ocean basins, it is clear that colonization of Atlantic turtles into the Indian Ocean occurred in recent evolutionary history (see discussion in [46]); but these events are rare, and likely only to happen when all of the conditions are just right. 

When looking at the results by regional genetic stock areas in the SWIO, some interesting conclusions can be drawn.

### 4.2. The North Area 

**Figure A1:**
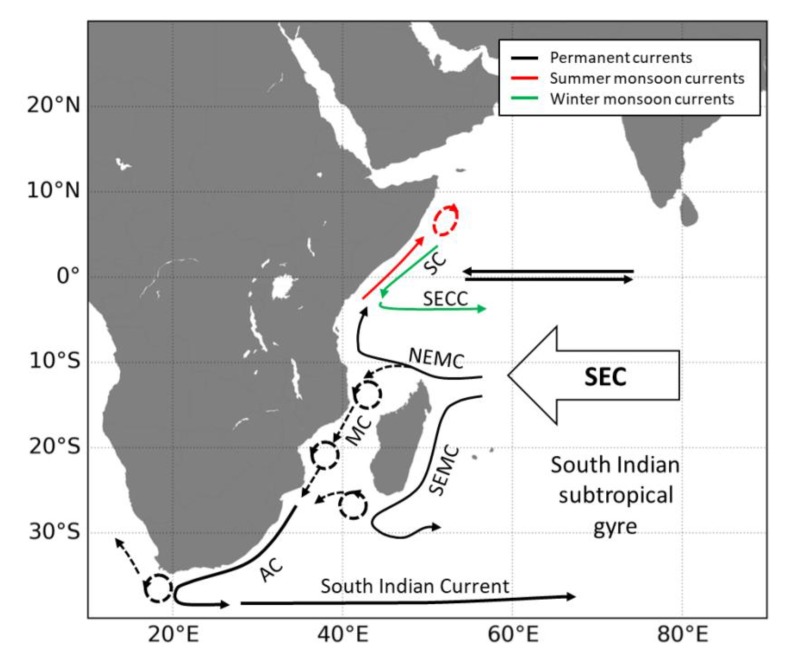
Schematic representation of the surface currents in the west Indian Ocean. Permanent currents are in black. Currents flowing during the summer monsoon are in red. Those flowing during the winter monsoon are in green. SC: Somali Current (reverses between the summer and the winter monsoon); SECC: South Equatorial Counter Current; SEC: South Equatorial Current; NEMC: Northeast Madagascar Current; SEMC: Southeast Madagascar Current; EACC: East African Coast Current; MC: Mozambique Current; Agulhas Current. Details of the whole Indian ocean circulation can be found in [54]. Ocean circulation is markedly different in the northern and southern Indian basins. In the southern hemisphere, circulation is rather stable throughout the year, being organized around an anticyclonic subtropical gyre, and driven by southeast trade winds in the tropics and westerlies in the middle latitudes. On the northern flank of the gyre, the South Equatorial Current (SEC) flows westward within a broad latitude range (sometimes up to 6°S, down to 25°S). It brings surface water all the way from Indonesia to the Madagascar east coast, where it splits into a northward branch (the Northeast Madagascar Current –NEMC) and a southward one (the Southeast Madagascar Current –SEMC). The NEMC flows past Cape Amber (the northern tip of Madagascar), and then feeds both the northward-flowing East African Coast Current (EACC) and the southward-flowing Mozambique Current (MC). The flow through the Mozambique Channel is characterized by an intense mesoscale activity, dominated by large anticyclonic eddies propagating southward along the Mozambique coastline. The associated mean flow is indeed southward, but the variability is large and northward transport events are not unusual (e.g., [93]). The SEMC flows southward along the East coast of Madagascar, and then becomes more turbulent, generating eddies that can either recirculate eastward around the South Indian subtropical gyre, or move westward towards the African coast, merging into the MC, and then the Agulhas Current (AC). As this current flows past the Cape of Good Hope, it can shed eddies that will then circulate into the South Atlantic Ocean, or retroflect into the Indian Ocean, and feed into the South Indian current. Circulation in the Northern hemisphere has a much stronger seasonal variability, governed by a monsoon regime with winds blowing from the southwest during summer, and reversing to the northeast during the winter monsoon. Consequently, the Somali Current (SC) flows northward during the summer monsoon. It can reach up to the Socotra Island, eventually forming some gyres. During the winter monsoon, the SC reverses, flowing southward down to about 3°S, where it meets the EACC. These two currents feed the seasonal eastward flowing South Equatorial Countercurrent (SECC). In the equatorial band itself, a singular phenomenon (not detailed here) causes surface currents to reverse direction (eastward/westward) four times a year.

The results from the MSA suggest that this region contributed very little (3–6%) to the genetic structure of juvenile green turtle in developmental sites further south (Figure 1; Table 2). This pattern is supported by four different arguments. First, the dispersal simulations suggest that particles from the North Area are dispersed westward by the dominant current system, being the South Equatorial Current (SEC), northward by the East African Coastal Current (EACC), and then eastward by the SECC [57,58], rather than south into the Mozambique Channel (Appendix A; Figure A1). Second, this study does not include any juvenile developmental sites from the North Area, the most important of which are found on the Amirantes Bank and at adjacent Desroches Atoll, all of which host extensive seagrass meadows and important foraging habitat for both adult [16] and juvenile [59] green turtles. Third, the genetic structure of nesting green turtles from the North Area is different from the other nesting sites of the SWIO, a structuring driven mainly by current dynamics and by the fact that nesters in the Amirantes group display a pattern of short distance post nesting migrations [39], supporting the hypothesis of limited genetic linkage between the Seychelles area and the rest of the SWIO. Fourth, although the Seychelles granitic and Amirantes groups together account for <3% of the total egg clutches produced in the Central and South Areas [37], Bourjea et al. [39] found preliminary evidence that the North Area shares haplotypes with green turtle nesting populations in the Chagos Archipelago, located north-east of the Seychelles, but considered part of the SWIO region [60], with an estimated 20,300 egg clutches laid annually [37]. Given that the Chagos Archipelago hosts relatively little seagrass habitat [61], more research is needed to determine the regional and spatial distribution of its green turtle juveniles. 

### 4.3. The Central Area

The Central Area, on the other hand, represented a significant source of juvenile green turtles for the developmental habitats sampled in the SWIO region. This region hosts by far the largest nesting populations of the SWIO [62], with almost 20,000 nesters annually. Results from the MSA showed that this breeding population made up the majority of juvenile turtles at all sampled developmental sites, with the exception of the two most southern sites at Europa and Juan de Nova (Figure 3). The “rookery centric” MSA also indicated that most hatchlings born in this region (60%) settle in developmental areas within the central region. Such auto-recruitment accords with the dispersal modeling mainly driven in this area by the Northeast Madagascar Current, and more particularly in the North of the Mozambique Channel, by a large stable anticlockwise gyre centered in the Comoro Basin [63] (Appendix A; Figure A1). Associated with abundant production of juveniles and propitious feeding grounds [64], this area could be considered robust in term of the recruitment of juveniles with genetic characteristics typical of the SWIO region [46]. Such a robustness is probably one of the main reasons that some nesting populations recover quickly and positively after past overexploitation of nesters. An example is Glorieuses Island that hosted very few clutches of green turtle in the 1970s [65,66], but nowadays, after 40 years of protection, hosts a nesting population of 1500–2500 nesters, with an annual rate of increase of 3.5% [67].

### 4.4. The South Area 

The three developmental sites in the South Area all showed a high proportion of juveniles originating from rookeries within the South Area. Being the southernmost nesting site for green turtles, and part of the southernmost foraging grounds of the SWIO for this species, the Europa atoll is known to be one of the most important nesting sites in the Indian Ocean [68], hosting more than 8000 nesters per year. Its contribution as a source of juveniles for the region may therefore be highly important. However, the “rookery-centric” MSA indicates a high level of auto-recruitment (64%, driven by the CM-A08 haplotype; Figure 2, Table S4), with a limited contribution (17%) to the Central Area. This pattern is corroborated by the dispersal simulations, with oceanic current flow in the Mozambique Channel dominated by a train of large anti-cyclonic eddies propagating southward, and having originated from within the Northeast Madagascar Current [69] (Appendix A; Figure A1). Furthermore, we expected a significant contribution from the Central and Northern Areas to the Europa developmental area; however, most juvenile turtles at Europa originate from local rookeries (most likely from Europa itself). Such a high percentage of auto-recruitment could be explained by three hypotheses. The first is that there is another unknown, important nesting site in the Indian Ocean dominated by the CM-A08 haplotype connected with the South Area; but this is unlikely, as all significant nesting sites are included in the present study. The second is that important gene flow is occurring from a rookery in the Atlantic largely dominated by CM-A08; but this is also unlikely, given oceanographic characteristics linking the Atlantic and Indian Oceans, as discussed above (see also the discussion above and in Bourjea et al. [46]). Such biogeographic and oceanographic boundaries are known to disrupt gene flow in many marine species [70]. The third is that juveniles found at Europa come from Europa itself. This is by far the more reliable hypothesis. Passive dispersion modeling of hatchlings from Europa indicates that water flow is expulsed from the South Mozambique Channel into (1) the Agulhas Current, and then to the South Atlantic, and (2) into the Agulhas Return Current, flowing back in the Indian Ocean along the Subtropical Convergence in cold waters (<15 °C); [71] (Appendix A; Figure A1). Hatchlings may then experience only a few potential foraging sites in the south of Madagascar and Mozambique, the Indian South African coast and Europa, all known to be foraging grounds for the green turtle [72], and then return preferentially to the few foraging grounds they have visited. Even if we expect high mortality due to cold water at dispersal, it is likely that the dispersal of green turtles may be driven by both passive drifting and active swimming to avoid such cold water, as shown by Gaspar and Lalire [31] and Lalire and Gaspar [5] for the leatherback turtle. 

### 4.5. The Case of La Réunion 

**Figure A8:**
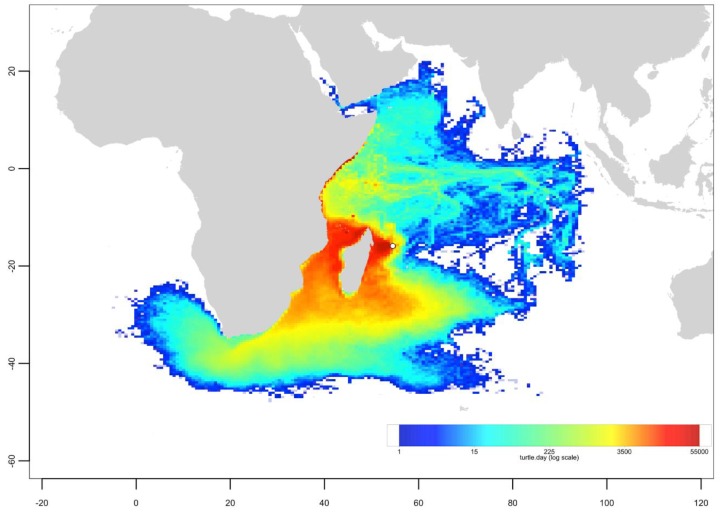
Tromelin dispersal map. One-year long trajectories of 10,000 particles released from a 0.25° × 0.25° areas located about 40 km off the main nesting beaches of Tromelin (with circle) from 2002 to 2006 (for a total of 50,000 particles). The color indicates the density of particles in number of turtles per day (log scale).

La Réunion represents a small and geographically isolated site from most foraging and nesting green turtles in the SWIO. The results of the MSA indicate that most turtles foraging at La Réunion originate from the Central Area (85%), with only small contributions from the North Area (4%) and the South Area (10%) (Figure 1, Table 2). However, this represents only a small fraction (4–14%) of the turtles being produced at the three regions (Figure 2). This low level of connectivity may be explained by the geographical isolation of La Réunion, and is supported by the dispersal simulations showing limited connectivity to all breeding populations except Tromelin (Appendix B, Figure A8). However, the current genetic structure does not allow for fine scale assessments beyond the three main genetic stocks. Future studies using additional genetic markers may increase genetic resolution, in order to answer whether or not Tromelin is the only site that contributes significantly to the La Réunion developmental site (see Section 4.7). 

La Réunion was a hotspot for nesting sea turtles in the seventeenth century [73], but intense human exploitation caused the population to collapse, and even with almost full protection since the 1990s, recovery has been very slow (a couple of nesters per year;) [74] compared to sites in the Central Area, such as Mohéli [62], Glorieuses [75] and Aldabra [76]. The same patterns of recovery are evident for foraging juvenile and adult turtles in the La Réunion coastal habitats. This population was not abundant, but regularly observed along the coastline in the 1990s, and then it significantly increased since then [41]. The same situation is observed for other islands of the Mascarene group, such as Mauritius [40] and Rodrigues [77]. Such low recovery of both nesting and foraging populations could be the consequence of the isolation of La Réunion from other nesting sites in the SWIO, due to oceanic currents dominated by the westward South Equatorial Current [78] (Figure 2 and Appendix B). Such isolation of the Mascarene group would interfere with the mixing of populations, as indicated by the low genetic diversity at the juvenile foraging grounds (Table 1), and low levels of gene flow from the North, Central and South Areas (Figure 2). This situation also indicates that the La Réunion nesting site and foraging grounds are more fragile, with a lower capacity for recovery than the nesting and foraging sites in the Central Area. 

### 4.6. Regional Connectivity Lessons from the SWIO

For large pelagic fishes that show cosmopolitan distribution, large population size, high fecundity, the production of numerous pelagic larvae, and the ability to easily migrate across an entire ocean, no structuring at the scale of the SWIO, or even at the scale of the Indian Ocean, was found (e.g., bigeye tuna [79] and swordfish [80]). Although characteristics of the sea turtle life cycles prevent direct comparison with fish or more general marine species models, several studies of coastal marine species have shown a number of patterns for different taxa, emphasizing the complexity of connectivity patterns, and its dependence on many factors, including life history traits and physical oceanography [81]. 

The isolation and low genetic diversity of the Mascarene group (e.g., La Réunion) vs. high connectivity within the northern Mozambique Channel have been shown for several species of coral (see review in [82,83]), seagrass [84], but also for several species of coral reef fishes showing various life traits and strategies, e.g., blue barred parrotfish [85], the blotcheye soldierfish [86], and the honeycomb grouper [87]. Muths et al. [86,87] analyzed samples of two species of fish from Europa and the Seychelles granitic islands, and showed that genetic structuring was more apparent in the South and North Areas than in the Central Area. Together these studies highlight the key role of oceanic currents in shaping the genetic structure of marine species across the SWIO. 

### 4.7. Limitations and Future Directions

The SWIO region shares haplotypes with both the Atlantic and the eastern Indian Ocean and the Western Pacific. This provides challenges for accurately estimating the stock origin, and complicates a confident assessment of contributions from these areas based on the global mtDNA genetic data alone. When considering all rookeries that share at least one haplotype with the foraging samples, the MSA included 38 genetic stocks spanning three ocean basins. This problem is caused by common and widespread haplotypes, such as CmP49, which is common in both the SWIO and throughout Asia-Pacific, as well as haplotypes CM-A08, which is common in the SWIO and throughout the South Atlantic. When included, stocks in these distant regions result in considerable uncertainty surrounding the estimates for these stocks. We excluded these distant rookeries from our study using sound biological reasoning based on geographical distance, ocean current patterns and genetics. Additional markers still targeting the maternal lineage, such as longer mtDNA d-loop sequences [88,89], whole mitogenome sequencing [90] and mtDNA STRs [91] may offer a better resolution and will most probably unravel links with distant stocks. Furthermore, the advances into genomics provides a fast and affordable way to discover and genotype thousands of genetic markers for a large group of individuals, including non-model species, such as marine turtles [92]. This will allow for an increasingly robust analysis of population structure, and may offer a better resolution and resolve issues related to common and shared haplotypes in future studies.

## Figures and Tables

**Table 1:** Frequencies of 13 mitochondrial DNA D-loop haplotypes (385 bp) found in eight juvenile Chelonia mydas foraging sites thoughout the Southwest Indian Ocean (SWIO). Also shown is haplotype (Hd) and nucleotide (π) diversity for each foraging site. Shaded area shows "orphan" haplotypes that have not yet been recorded at a nesting site.

					Haplotype ID												
**Location**	**Coordinates**	**π**	**Hd**	**n**	CM-A08	CmP49	CmP87	CmP40	CmP75	CmP57	CmP47	CmP32*	CmP83*	CM-A46*	CmI11	CmI9	CmP176
Kenya	3°30’S–40°11’E	*0.0223*	*0.4966*	**67**	6	47	1	1	2	1	6	2	-	-	-	1	-
Aldabra	9°41’S–46°21’E	*0.0154*	*0.3333*	**47**	-	38	-	-	1	-	5	-	-	-	-	1	2
Glorieuses	11°58’S–47°29E	*0.0328*	*0.6874*	**31**	3	17	1	-	-	1	7	1	-	-	-	1	-
Mayotte	12°98’S–45°19’E	*0.0241*	*0.4778*	**53**	6	37	-	-	3	-	4	3	-	-	-	-	-
west Madagascar	18°25’S–43°55’E	*0.0285*	*0.5585*	**33**	9	19	-	-	2	-	1	1	-	-	1	-	-
Juan de Nova	17°05’S–47°72’E	*0.0339*	*0.6724*	**24**	12	8	-	-	2	-	-	1	1	-	-	-	-
Europa	22°34’S–40°37’E	*0.0326*	*0.5946*	**38**	22	10	1	-	1	-	3	-	-	1	-	-	-
La Réunion	21°05’S–55°22’E	*0.0280*	*0.5005*	**65**	6	44	-	-	-	-	13	1	-	-	-	-	1
**Total**				**358**	**64**	**220**	**3**	**1**	**11**	**2**	**39**	**9**	**1**	**1**	**1**	**3**	**3**

* Haplotypes found only at rookeries outside the SWIO; CmP32 (Micronesia), CmP83 (Western Australia), CM-A46 (Acension Island).

**Table 2:** Results from the “many-to-many” mixed stock analysis (MSA) using the package “mixstock” in R for eight green turtle (Chelonia mydas) feeding grounds in the Southwest Indian Ocean. Shown are the locations, sample size (n), number of orphan haplotypes (#orphan (total individuals)), percentage of orphan haplotypes (orphan), mean contribution from each of the three SWIO nesting stocks as well as Cocos (Keeling) Island (CKI) and 95% confidence interval.

				North		Central		South		CKI	
**Location**	**n**	**# orphan**	**% orphan**	**mean**	95% CI	mean	95% CI	mean	95% CI	mean	95% CI
Kenya	67	1(1)	1%	3%	(0–12)	85%	(70–95)	11%	(2–22)	1%	(0–5)
Aldabra	47	2(3)	6%	4%	(0–19)	92%	(73–99)	3%	(0–11)	1%	(0–6)
Glorieuses	31	1(1)	3%	6%	(0–28)	79%	(45–96)	14%	(1–31)	2%	(0–10)
Mayotte	53	0(0)	0%	4%	(0–16)	80%	(58–94)	15%	(3–30)	2%	(0–7)
west Madagascar	33	1(1)	3%	4%	(0–19)	56%	(28–79)	39%	(18–65)	1%	(0–7)
Juan de Nova	24	0(0)	0%	4%	(0–16)	24%	(2–52)	71%	(43–94)	1%	(0–16)
Europa	38	0(0)	0%	5%	(0–17)	20%	(1–42)	74%	(53–91)	1%	(0–6)
La Réunion	65	1(1)	2%	4%	(0–19)	85%	(63–96)	10%	(1–21)	1%	(0–9)

**Table 3:** Particle simulation release information. Table showing the location of release sites for the particle simulation as well as the release period corresponding with the peak hatching emergence for each population.

Nesting Area	Long (°E)	Lat (°S)	Release Period
Seychelles granitics Group	55.6	4.46	August 1st to October 31
Seychelles Amirantes Group	53.4	5.63	August 1st to October 31
Glorieuses Island	47.3	11.57	1st June to 31 August
Mayotte Island	45.15	12.13	1st July to 31 September
Mohéli Island	43.73	12.32	1st June to 31 September
Tromelin Island	54.52	5.89	1st September to 31 November
Europa Island	40.36	22.37	1st January–31 Marsh

**Figure A2:**
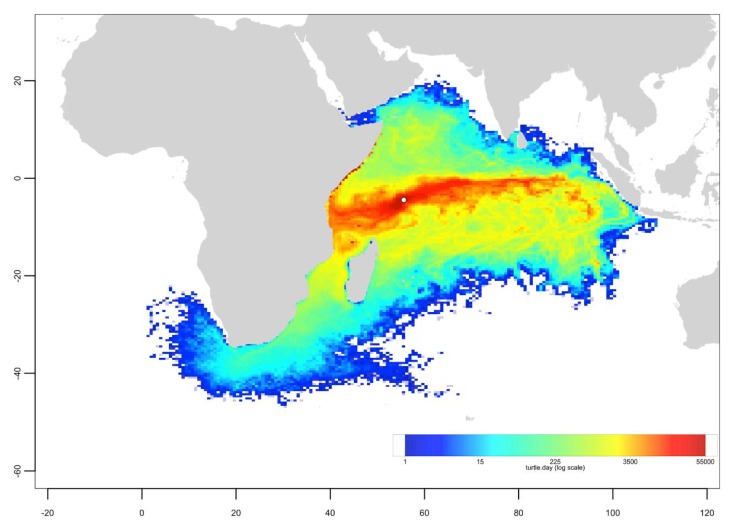
Granitic islands group dispersal map. One-year long trajectories of 10,000 particles released from a 0.25° × 0.25° areas central to Granitic Group (with circle) from 2002 to 2006 (for a total of 50,000 particles). The color indicates the density of particles in number of turtles per day (log scale).

**Figure A3:**
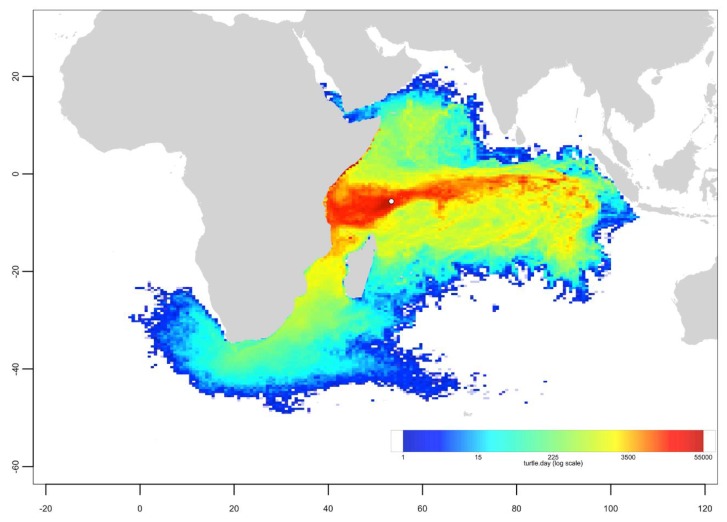
Amirantes group dispersal map. One-year long trajectories of 10,000 particles released from a 0.25° × 0.25° areas central to the Amirantes group (with circle) from 2002 to 2006 (for a total of 50,000 particles). The color indicates the density of particles in the number of turtles per day (log scale).

**Figure A4:**
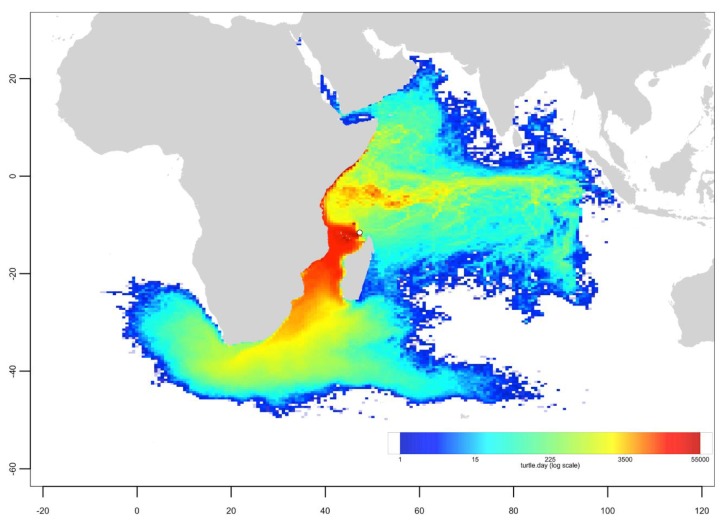
Glorieuses dispersal map. One-year long trajectories of 10,000 particles released from a 0.25° × 0.25° areas located about 40 km off the main nesting beaches of Glorieuses (with circle) from 2002 to 2006 (for a total of 50,000 particles). The color indicates the density of particles in number of turtles per day (log scale).

**Figure A5:**
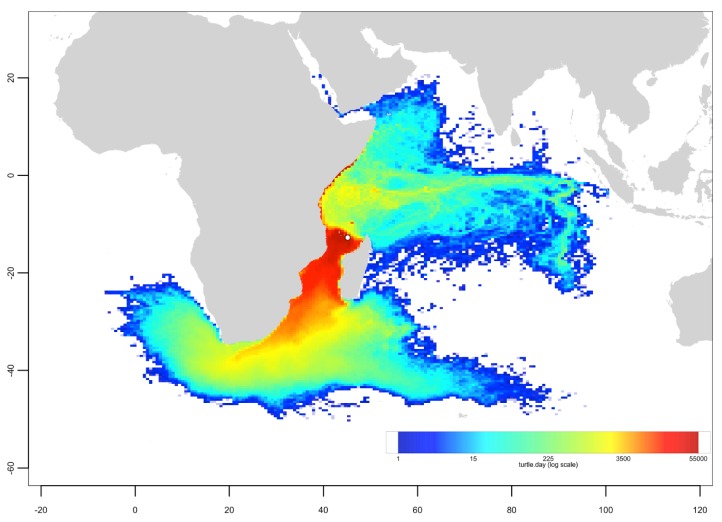
Mayotte dispersal map. One-year long trajectories of 10,000 particles released from a 0.25° × 0.25° areas located about 40 km off the main nesting beaches of Mayotte (with circle) from 2002 to 2006 (for a total of 50,000 particles). The color indicates the density of particles in number of turtles per day (log scale).

**Figure A6:**
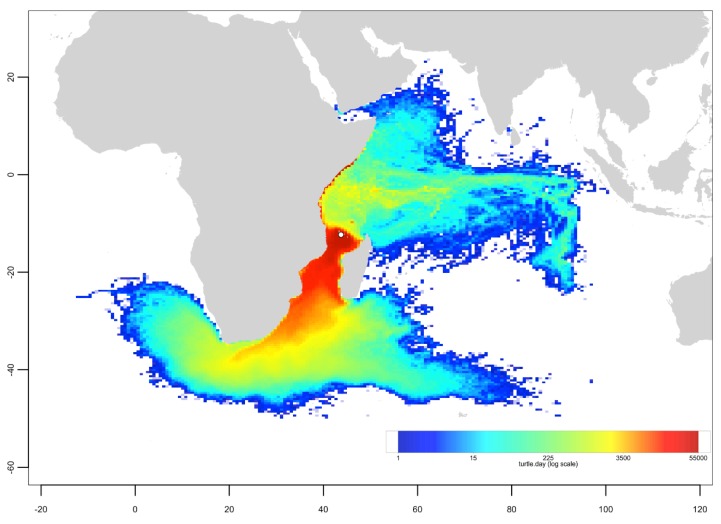
Mohéli dispersal map. One-year long trajectories of 10,000 particles released from a 0.25° × 0.25° areas located about 40 km off the main nesting beaches of Mohéli (with circle) from 2002 to 2006 (for a total of 50,000 particles). The color indicates the density of particles in number of turtles per day (log scale).

**Figure A7:**
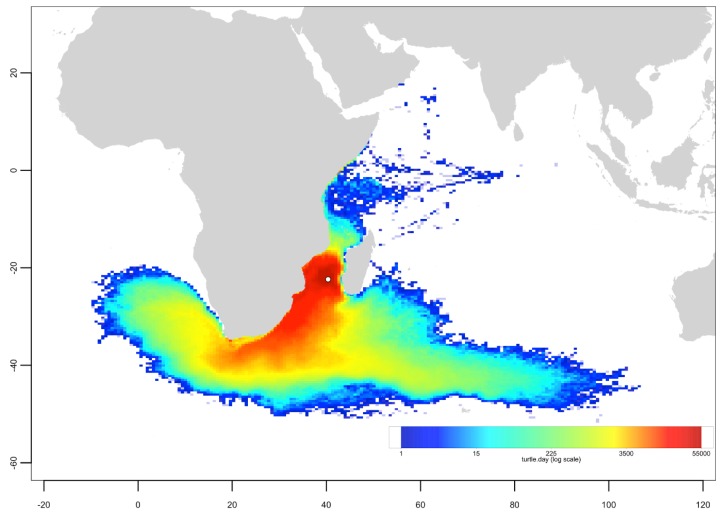
Europa dispersal map. One-year long trajectories of 10,000 particles released from a 0.25° × 0.25° areas located about 40 km off the main nesting beaches of Europa (with circle) from 2002 to 2006 (for a total of 50,000 particles). The color indicates the density of particles in number of turtles per day (log scale).

**Figure A9:**
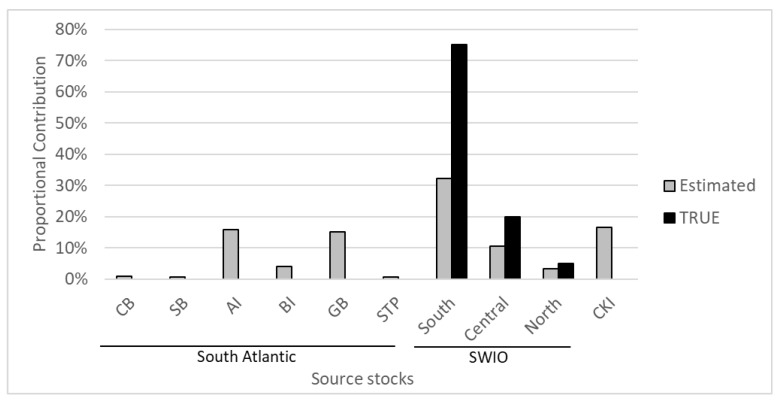
Simulated MSA. Graphs showing the proportional contributions estimated by BAYES (gray) from ten genetic stocks across the south Atlantic (Central Brazil, CB; South Brazil, SB; Ascension Is, AI; Bioko, BI; Sao Tome and Principe, STP), Southwest Indian Ocean (South; Central; North), and Cocos (Keeling) Islands, CKI to a hypothesized foraging aggregation. The simulated “true” value (black) of the composition represents 75% from the South, 20% from the Central, and 5% from the North Areas of the SWIO, with no contribution from Atlantic stocks and Cocos (Keeling) Islands.

## Supplementary Materials

The following are available online at https://www.mdpi.com/2073-4425/11/3/278/s1, Figure S1. Haplotype network. Haplotype network of 19 Chelonia mydas mtDNA haplotypes (385 bp) found at three genetic stock regions in the Southwest Indian Ocean. Also shown are “orphan” haplotypes that have been identified in feeding sites, but not at any rookery; Figure S2. Global MSA. Graphs showing the proportional contributions from 38 genetic stocks that share at least one haplotype with the eight developmental sites used in this study. The 38 genetic stocks spanned three major ocean basins, and included stock in the Atlantic (*Central Brazil, CB; South Brazil, SB; Ascension Is, AI; Bioko, BI; Sao Tome & Principe, STP*), Southwest Indian Ocean (*South; Central; North*), Northwest Indian Ocean (*Saudi Arabia, SA; Oman, OM; Arabian Gulf, AG*), Central and East Indian Ocean (*Cocos (Keeling) Islands, CKI; West Java, WJ; Ashmore Reef, AR, Scott Reef/Browse Is, SRBI; Northwest Self, NWS; Coburg Peninsula, CP*), Southeast Asia (*Gulf of Carpentaria, GOC; Sangalaki, SAN; Sipidan, SIP; Turtle Islands, TI; Sarawak, SAR; Peninsular Malaysia; PMAL, east Taiwan, ETAI; Central Ryukus, CRY*) and the Western Pacific (*Ogasawara, OGA; Mariana Islands, CNMI; Mironesia, MIC; Palau, PAL; Papua New Guinea, PNG; Marshall Islands, RMI; Great Barrier Reef, nGBR, sGBR; Coral Sea, CS; New Caledonia, NC; Vanuatu, VAN; American Samoa, AMS*). Table S1. Haplotype frequencies. Haplotype frequencies for the four green turtle (*Chelonia mydas*) breeding stocks (and individual rookeries), and eight foraging sites used in the mixed stock analysis. Also shown are estimated population sizes (annual number of nesting females), three letter country code, latitude and longitude and references used for the baseline data; Table S2. Sampling information. Table showing the location of eight green turtle developmental areas sampled in this study, as well as samples size, sampling year and the mean curved carapace length (CCL), with standard deviation (SD); Table S3. Developmental area centric MSA. The proportional contribution of four green turtle genetic stocks to eight different developmental areas in the Southwest Indian Ocean using mixed stock analysis. The results show a comparison of two different methods: a “many to one” method using the software Bayes, and a “many to many” method using the R-package mixstock. The tables and graphs show the mean contribution as well as the lower and upper 95% credibility intervals; Table S4. Rookery centric MSA. The proportional of green turtles from each of the three SWIO genetic stocks going to eight different developmental areas in the Southwest Indian Ocean, using mixed stock analysis. Results are also summarized by region, and shown as the mean contribution, as well as the lower and upper 95% credibility intervals.
