# Supplementary figures and images for: Seascape Genetics and the Spatial Ecology of Juvenile Green Turtles
Source: Genes (Basel). 2020 Mar 5;11(3):278. doi: 10.3390/genes11030278 (PMC7140902; doi:10.3390/genes11030278)

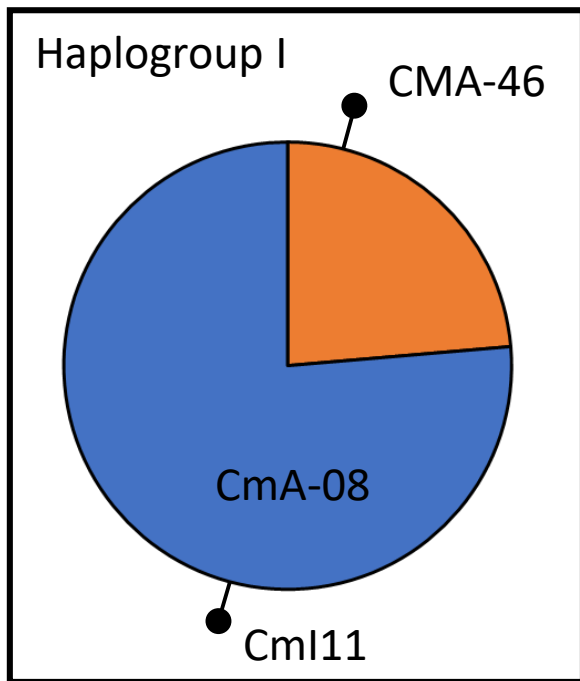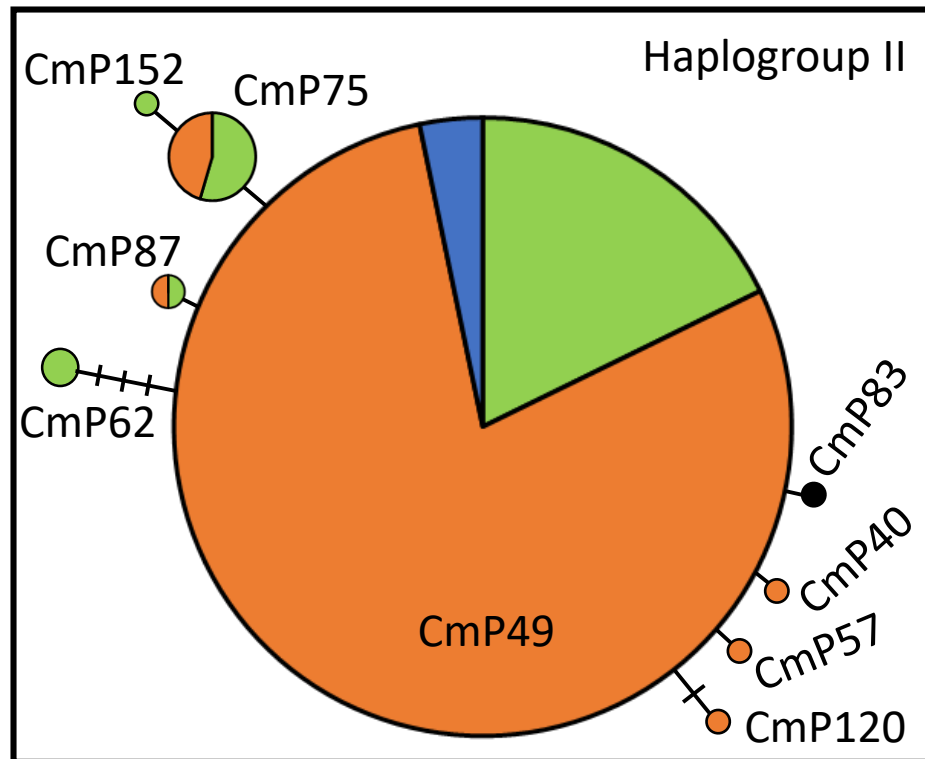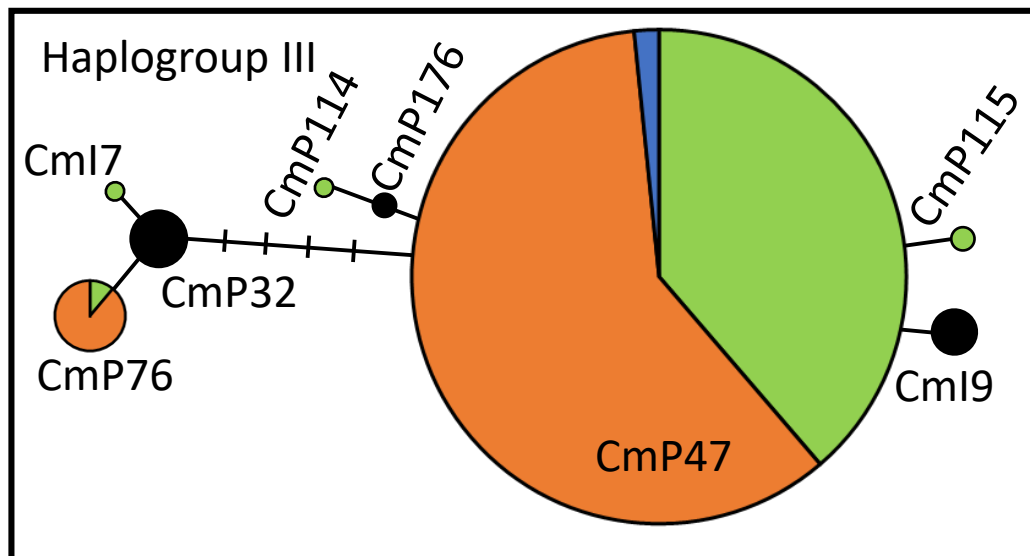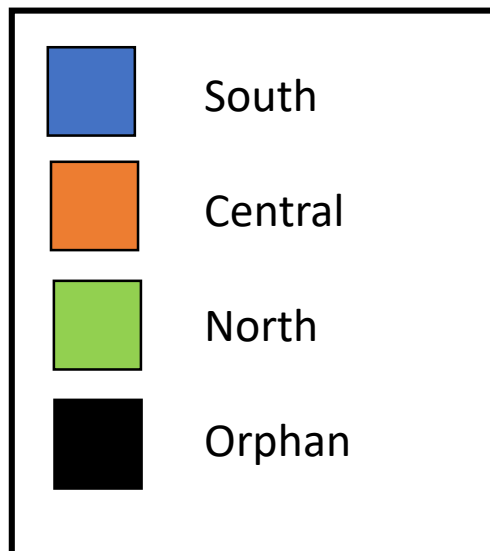

Supplement: Supplementary file 1 [file genes-11-00278-s001.zip › genes-721195-supplementary/Figure S1.pdf]

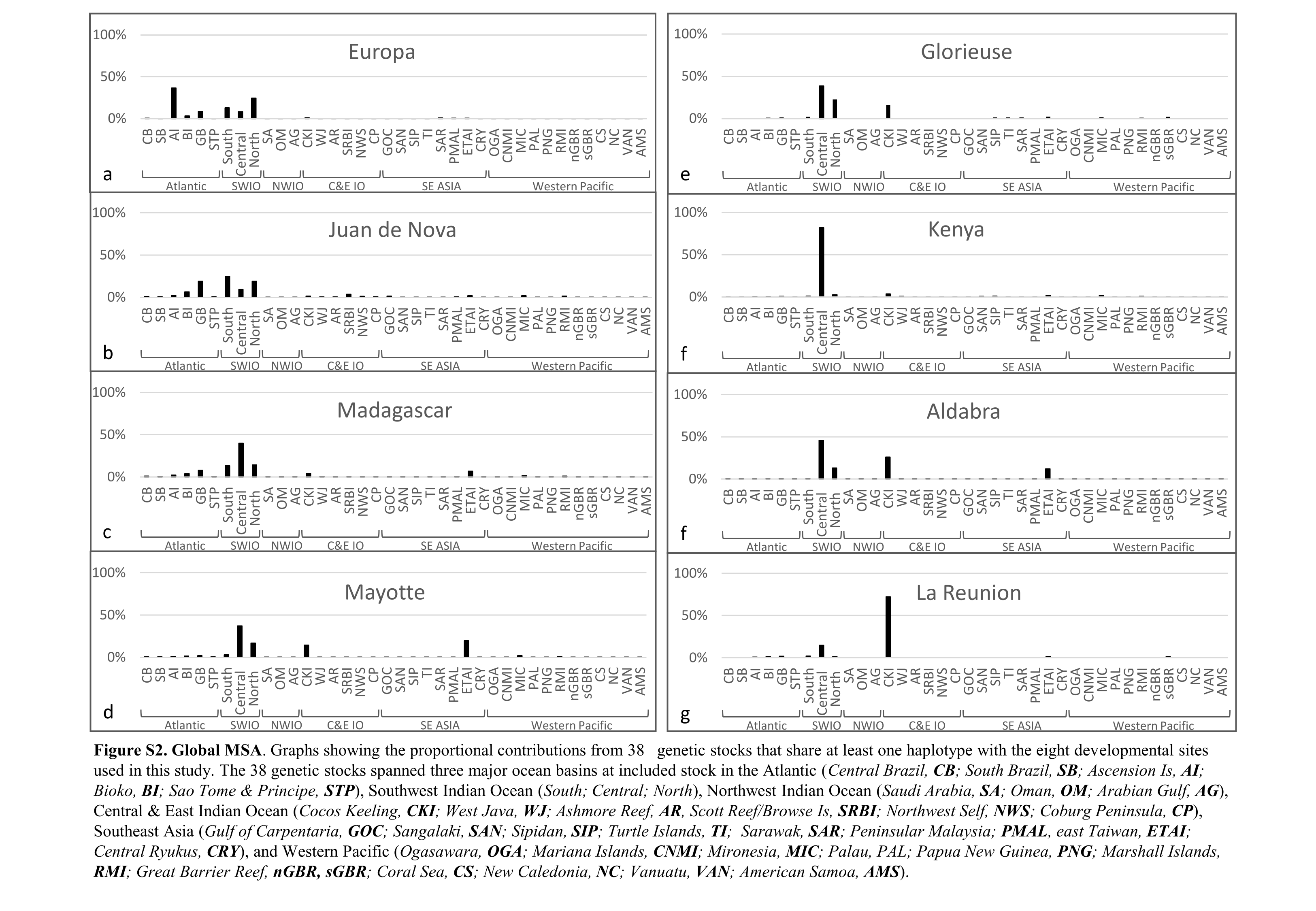

Supplement: Supplementary file 1 [file genes-11-00278-s001.zip › genes-721195-supplementary/Figure S2_HD.png]
